# ALLERGIC BRONCHOPULMONARY ASPERGILLOSIS WITH ASPERGILLUS SINUSITIS-‘9’ YEAR OLD BOY

**DOI:** 10.4103/0970-2113.45282

**Published:** 2008

**Authors:** Pratima Das, Rakesh Arya, S. K. Shrivastava

**Affiliations:** Department of Respiratory Medicine, Gandhinagar Hospital, CCL, Ranchi

**Keywords:** ABPA, Aspergillus Sinusitis

## Abstract

A case of Broncho Pulmonary Aspergillosis with Aspergillus Sinusitis was diagnosed in a nine year old boy after clinical and immunological investigations. He responded well after treatment with steroid and itraconazol.

## INTRODUCTION

Allergic bronchopulmonary aspergillosis (ABPA) is an immunologically complex disease and the patient elicit Type I and Type III hypersensitivity reaction to Aspergillus Allergens / Antigens. ABPA is often encountered in patients of Bronchial Asthma, Cystic Fibrosis, Sinusitis, Rhinitis etc. Many a times it causes a diagnostic dilemma as the clinical features mimic tuberculosis. Early detection and diagnosis of ABPA is important in view of preventing lung damages, severity of asthma, misdiagnosis of patients for tuberculosis etc. ABPA in a child should be diagnosed early to prevent Bronchiectasis in adulthood.

We are reporting a case of ABPA with Aspergillus Sinusitis in a 9 year old boy who was managed with Antifungal and Steroid. The management modality was initiated to prevent complications.

## CASE REPORT

A 9 year old boy presented with low grade fever chest pain, breathlessness, thick yellow nasal discharge. He was having history of taking treatment for Allergic Sinusitis followed by Br.Asthma since 3 years of his age. When he was 4 years old, he was treated with ATT on the basis of having small opacities in the lung (radiologically) although he was sputum negative.. He had also complained of expectoration with brown colour sputum. He was treated with antibiotics, antihistminics, corticosteroid (both nasal & inhaled) & bronchodialater several times since his childhood but the symptoms remained unabated.

On seeing the patient it was found that he had temperature 96° C (axilary), pulse rate was 84/min,.his respiratory rate was 16/min, and his blood pressure was 110/60 mm of Hg. The physical examination revealed B/L rhonchi & his peripheral blood examination showed easinophilia 8%, Hb 11% | :- ESR : 12 mm / hour, WBC count 8400/cm, skin prick test for Aspergillus was positive ( 10 × 6 mm ), total IgE was 4838.5 / IU, aspergillus Specific IgE Allergy score was 3.2 ( 0.00 – 0,50 )Class II. X-Ray chest (PA) showed fleeting opacities present. X-Ray PNS showed Pansinusitis with thick mucus plug in frontal and Maxillary sinuses. ENT specialist was consulted to exclude nasal Polyp. The patient refused for nasal endoscopy hence smear was taken from maxillary sinus through sinoscopy. The culture of the nasal smear showed fungal hyphae. Pulmonary function tests were FEV1, –53.9%, FVC 64.1%and FEV1 % −73.6 %, HRCT thorax showed no Bronchiectasis. HRCT paranasal sinuses was not done as patients parents refused it. Based on the above finding the diagnosis which was arrived was “Broncho Pulmonary Aspergillosis with Aspergillus Sinusitis” and the treatment was started with prednisolone 0,5 mg / kg / day and Itrakonazol and Steroid nasal Spray. In the first follow up visit after one month, the patient showed improvement. Total Serum IgE was 2925.4/IU. The Prednisolone dose was decreased in the second monthly visit. There was no complaints and total IgE was 1878.4/IU . Pulmonary function test was normal. X-Ray PNS showed improvement and X-Ray chest showed no opacities. Itraconazol was stopped after 4 months. In the fourth visit IgE was 656 IU/ ML and again Prednisolone dose was reduced. On the fifth monthly visit, patient was found to be normal and IgE had maintained a plateau. Prednisolone was reduced to 7.5 mg / day for one month then subsequently it was reduced to 5.mg per day for another month. Following which it was further reduced to 5 mg alternate day & then the treatment was stopped. The patient was seen after 8 months since the initiation of treatment where it was found that he had no complaints and total Serum IgE was 650 IU/ML. PFT was normal. X-Ray chest (PA) and X-Ray PNS were normal.

## DISCUSSION

Aspergillus is a fungus with a worldwide distribution. This fungus is found in decomposing organic matter and can colonize walls and ceilings where water seepage has occurred. Out of 180 species of aspergillus, aspergillus fumigates. is one of the commonest fungus which causes ABPA & severe destructive lung disease. Early diagnosis is essential to prevent the development of end stage lung fibrosis. Imaging and immunological techniques have been crucial in the early diagnosis of the disease. ABPA is often present for years before diagnosis. Lungs and sinuses are the common sites involved & effected by ABPA. Most of the ABPA cases are usually undiagnosed & treated with anti tuberculosis drugs. Patients with ABPA in childhood if not diagnosed early but treated with ATT usually develop Bronchiectasis & scarring in adulthood & ultimately they landup in taking treatment for post tubercular bronchiectasis.

The case in discussion has developed ABPA since childhood. The fleeting opacities was treated with ATT and aspergullus sinusitis was treated with antiallergic drugs. He was also taking irregular treatment for bronchial asthma. This case responded very well to Itraconazol & oral steroid. Itraconazol & steroid combination are beneficial for such patients.

The purpose of reporting this case is - to highlight the unawareness in periphery about ABPA & its complications moreover ABPA & Aspergillus sinusitis in a 9 years old boy is a rare presentation.
